# 

**Published:** 1892-04-30

**Authors:** 


**Notices of Births, Deaths, and Marriages**, are inserted in this column at a charge of 2s. 6d. for an announcement not exceeding four lines.

### NOTICE TO CORRESPONDENTS.

All MS., Letters, Books for Review, and other matters intended for the Editor should be addressed The Editor, The Lodge, Porchester Square, London, W.

The Editor cannot undertake to return rejected M.S., even when accompanied by stamped directed envelope.

**Notice to Advertisers and Subscribers.**

All Advertisements, Orders for Copies of The Hospital, and Business Communications should be addressed to The Publisher, *not to the Editor*, The Hospital, 140, Strand, W.C.

The Cost of Subscription, post free, is as follows:—Per week, 2½d.; per quarter, 2s. 6d.; per half-year, 5s.; per annum, 8s. 6d. Foreign:—Per annum, 10s. 8d.; payable, in all cases, in advance.

**"Burdett's Hospital Annual," 1891—1892.**

Third year of publication. 600 pp. Boards, gilt lettering. A most complete guide to British and Colonial Hospitals, Dispensaries, Nursing and Convalescent Institutions, and Asylums. Price 3s. 6d. Published by The Scientific Press (Limited), 140, Strand, W.C.

**NOTICE. The Index for Vol. X. (ending September, 1891), is on sale at the Office of this Journal, price 2½d., post free.**

### Scraps and Gleanings.

The death of William McCook, aged 105 years, is reported from Ballymoney, County Antrim.

Dr. Sinclair White has been appointed Honorary Surgeon of the Sheffield Public Hospital and Dispensary.

The new wing of the Kent County Ophthalmic Hospital, Maidstone, was opened on the 22nd inst. by the Earl of Darnley.

At a meeting held recently at Cardiff it was proposed to expend £800 for the erection of one of Humphrey's iron hospitals, to accommodate 24 patients.

The Committee of the Luton Cottage Hospital have decided to enlarge it, and to provide for eleven extra beds. It is estimated that the total cost of building, &c., will amount to £6[illegible]0.

The accounts of the Nottingham General Hospital for the year ending March 1st show that the total receipts amounted to £9,562 12s. 10d., and the expenditure to £9,514 13s. 2d.

During last year 16,935 cases were treated at the Leicester Infirmary. One of the great features of the work done consisted in dealing with accidents, of which there were 5,983 cases last year, an average of 16½ per day.

Sir John Gorst has officially announced that the Treasury are prepared to grant £40,000, in annual sums of £5,000, for the extension of Aberdeen University, on condition that a like amount is raised by local subscription.

The Directors of the Edinburgh Royal Hospital for Sick Children have appointed Mr. John Wheeler Dowden, M.B., C.M., and Mr. Robert D. Clarkson, B.Sc., M.B., C.M., to be resident physicians for six months from May 1st.

The ladies' street collection of the Hospital Saturday Fund will be made on July 16th, and the Council appeal for the assistance of ladies of the metropolis in superintending the collecting boxes on behalf of the London charities.

Colonel Williams, of Bridehead, near Dorchester, a Director of the London and South-Western Railway Company, has presented to the Dorset County Hospital one thousand pounds, in memory of his father, the late Mr. Robert Williams.

The total number of patients treated during the past year at the Worcester Dispensary was 9,381. The amount collected from the members of the Provident Branch was £1,422 5s. 6d., an increase of £22 5s. 10d. on the previous year.

By the death of Mrs. Sharp, of Row, Dumbartonshire, the Victoria Infirmary, at Glasgow, succeeds to the whole estate of her first husband, Mr. Couper, paper maker, of Cathcart, Glasgow. The amount is believed to be between thirty and forty thousand pounds.

At the annual meeting of the Leamington Provident Dispensary it was reported that 14,189 consultations were held at the dispensary, and 14,945 visits were paid to patients at their own homes. Owing to an epidemic of influenza, the expenditure exceeded the income by £30 12s. 7d.

In Westphalia a colony of epileptics, engaged in agricultural and other pursuits, has proved to work well. During their periodical fits, the patients receive every care and attention. It has been proposed by some medical specialists to start an institute of the same kind near the Metropolis.

During the eight years that the Cottage Home for Convalescent Children, Helensburgh, has been opened, 1,020 children have been received into it. During the summer months accommodation for 12 poor children is provided; during the winter months the number is reduced to 10.

The authorities in Paris have hit on the plan of taxing gambling stakes for the benefit of the "Assistance Publique." £28,000 derived from this source has been applied to the construction of a new consumption hospital near Paris, while the surplus of £4,000 has been given to other charitable purposes.

At a meeting held recently at Horsham for the purpose of electing the governing body of the Cottage Hospital, which is almost completed, the chairman announced that the amount already given and promised towards it amounted to £1,740 1s. 2d. In addition, £13 9s. 6d. had been received from the bank as interest.

At a meeting of the fruit porters and labourers, held recently at Liverpool in support of the Hospital Saturday Fund, the Mayor stated that if all the working men of the city would only contribute one penny a week it would represent in round numbers somewhere about £10,000 per annum towards the support of the hospitals.

The additions made recently to the Rotherhithe Infirmary consist of a new male block, with separation wards, day-rooms, and duty-rooms for 177 inmates; new female block for 96 inmates, nurses' home for 40 nurses; the enlargement of the laundry, kitchen, receiving wards, new offices, mess-room, and a new mortuary and post-mortem room.

One of the two most remarkable golden wedding presents to be given the King and Queen of Denmark on May 2nd is a golden crown of wheat-ears and clover, bought with the pennies of 100,000 school children, bearing the inscription: "The children of Denmark have woven this crown for the occasion of the golden wedding of King Christian and Queen Louise on May 26th, 1892."

Italy has started a hospital at Massowah. The hospital enjoys the patronage of His Majesty King Humbert, who has shown his interest in it by a grant of 5,000 lire (£200). There are many reasons why Italy should strengthen her foothold in those parts—the adaptability of her settlers to the climate, as already shown in the *Lancet*, giving her an advantage over other European competitors in the opening up of the Dark Continent.

### The Student of Medicine.

[All communications for this Supplement should be addressed to The Editor of The Hospital, at 140, Strand, with the words "Students' Column," written in the left hand top corner of the envelope.]

### APPOINTMENTS.

Barrs, A. G., M.D.Edin., M.R.C.P.Lond., formerly Assistant Physician, has been appointed Physician to the General Infirmary at Leeds. Bone, John W., M.B., C.M.Edin., appointed Senior House Surgeon to the Clayton Hospital and General Dispensary, Wakefield. Cant, William John, L.R.C.P., M.R.C.S., L.S.A., appointed Honorary Consulting Surgeon to the Lincoln General Dispensary. Clemow, George E., M.B., C.M.Edin., appointed House Surgeon to the Infirmary for Children, Myrtle Street, Liverpool. Duffus, George, M.B., C.M.Aber., appointed Medical Superintendent of the Brook Villa Asylum, West Derby, Liverpool. Eager, T. C., L.R.C.P., M.R.C.S., L.M., L.S.A., appointed Medical Officer to the Woking Post Office. Field, F. A., L.R.C.P.Lond., M.R.C.S., appointed Resident Surgeon to the Bedford General Infirmary and Fever Hospital. Gibbon, John George, B.A., M.B., B.Ch.Univ.Dub., appointed Resident Medical Officer to Grey's Hospital, Maritzburg, Natal, S.A. Hamilton-Whiteford, C., M.R.C.S.Eng., L.R.C.P.Lond., appointed Assistant House Surgeon to the South Devon and East Cornwall Hospital, Plymouth. Hamilton, William Cope, L.R.C.S., L.R.C.P.Irel., appointed House Surgeon to the Steevens's Hospital, Dublin. Jacob, Ernest H., M.A., M.D.Oxon., formerly Assistant Physician, has been appointed Physician to the General Infirmary at Leeds. Marsh, Frank, M.R.C.S., L.R.C.P.Lond., D.P.H.Camb., appointed Assistant Surgeon to the Birmingham and Midland Ear and Throat Hospital. Mason, George A., M.A., M.B., B.C.Cantab., appointed House Physician to the City of London Hospital for Diseases of the Chest, Victoria Park, E. Morgan, Rees, L.R.C.P.Edin., L.F.P.S.Glas., appointed Deputy Coroner for the East Division of Carmarthenshire. Reid, A. G., M.B., C.M.Edin., appointed House Surgeon to the Rotherham Hospital and Dispensary, vice L. J. Weatherbe, M.B., resigned. Rhodes, James, M.R.C.S., appointed Medical Officer of the Workhouse of the Glossop Union. Rowlands, F. Mortimer, B.A., M.B., B.C.Cantab., M.R.C.S., L.R.C.P.Lond., appointed Resident Medical Officer to the Workhouse Infirmary, Birmingham. Shaw, J. Hepworth, M.R.C.S., L.R.C.P.Lond., M.B., Ch.B. (Vict.), appointed Assistant Surgeon to the Liverpool South Dispensary, vice Dr. Molyneux, resigned. Watkins, D. J. G., B.A.Cantab., M.R.C.S., L.R.C.P., appointed House Surgeon to the Lincoln County Hospital. Wolverson, Thomas, L.R.C.P.Edin., M.R.C.S., appointed Surgeon to the Wolverhampton Police Force.

### VACANCIES.

### Resident Medical Officers.

The following vacancies are announced this week, the amount of salary in each case being given after the name of the institution:

General Hospital, Nottingham, Assistant House Physician—Board, &c.

Holloway and North Islington Dispensary, Resident Medical Officer—Salary commencing £120 per annum, with unfurnished house, gas, and coals, and £20 allowed for servant.

London Society for Promoting Christianity amongst the Jews, fully qualified Medical Man as Medical Missionary, under 30 years of age—Salary £250 per annum, with unfurnished lodgings.

Manchester Hospital for Consumption and Diseases of the Chest and Throat, Resident Medical Officer—Salary £60 per annum, with board, &c.

Rotherham Hospital, Rotherham, Assistant House Surgeon—Rooms, &c. Appointment for six months.

Sussex County Hospital, Brighton, Assistant House Surgeon; doubly qualified; unmarried, and under 30 years of age—Salary £30 per annum, with board, &c.

University College Hospital London, Resident Medical Officer.

West Norfolk and Lynn Hospital, King's Lynn, House Surgeon and Secretary—Salary £80, rising to £100 per annum, with board, &c.

### Non-Resident Medical Officers.

Brighton Throat and Ear Hospital, 23, Queen's Road, Brighton, Non-Resident House Surgeon—Salary at the rate of £50 per annum.

Charing Cross Medical School, Lecturer on Biology.

Chelsea Hospital for Women, Fulham Road, S.W., Clinical Assistant.

General Hospital, Birmingham, Honorary Physician.

King's College, London, Curator of the Museum.

St. John's Hospital for Diseases of the Skin, Leicester Square, Honorary Assistant Medical Officer to the Convalescent Branch, Bellgarth, Temple Fortune, Finchley Road, N.W.

Wolverhampton Hospital for Women, Lady Dispenser, thoroughly qualified—Salary £[illegible]5 per annum.

### THE STUDENT OF MEDICINE—(*continued*).

### PASS LISTS.

#### University of Durham.

First Examination for the Degree of Bachelor in Medicine, April, 1892.

The following candidates have satisfied the Examiners :—

Elementary Anatomy and Physiology, Chemistry with Chemical Physics, and Botany with Medical Botany.—First Class Honours: E. R. Kendall, College of Medicine, Newcastle-upon-Tyne. Pass List: B. Addenbrooks, Queen's College, Birmingham; S. Barker, Owens College, Manchester; W. L. T. Goodridge, Guy's Hospital; A. H. Hobbs, College of Medicine, Newcastle-upon-Tyne; W. R. Kingdon, College of Medicine, Newcastle-upon-Tyne; H. H. Lawrence, College of Medicine, Newcastle-upon-Tyne; W. H. Rowell, College of Medicine, Newcastle-upon-Tyne.

Elementary Anatomy and Physiology.—P. L. Armstrong, College of Medicine, Newcastle-upon-Tyne; H. R. Battiscombe, College of Medicine, Newcastle-upon-Tyne; A. B. R. Body, College of Medicine, Newcastle-upon-Tyne; W. G. Cook, College of Medicine, Newcastle-upon-Tyne; W. G. Fell, College of Medicine, Newcastle-upon-Tyne; G. W. Harbottle, College of Medicine, Newcastle-upon-Tyne; J. E. Harper, Queen's College, Birmingham; T. H. Urwin, College of Medicine, Newcastle-upon-Tyne; W. H. Whitehouse, Queen's College, Birmingham.

Chemistry with Chemical Physics, and Botany with Medical Botany.—A. Bryans, College of Medicine, Newcastle-upon-Tyne; F. A. Cooke, St. Mary's Hospital; H. H. Gourley, University, Edinburgh; V. Graham, St. Thomas's Hospital; L. Harman, St. Thomas's Hospital; J. S. Manford, College of Medicine, Newcastle-upon-Tyne; R. A. Mills-Roberts, College of Medicine, Newcastle-upon-Tyne; W. A. H. Waite, Yorkshire College, Leeds; P. Withers, Owens College, Manchester; H. T. M. Whitling, St. Bartholomew's Hospital.

Chemistry with Chemical Physics.—M. F. Cahill, L.R.C.P., L.R.C.S.I., Medical School, Catholic University, Dublin; L. C. E. Calthrop, M.R.C.S.Eng., L.R.C.P.Lond., London Hospital; F. W. Clark, M.R.C.S.Eng., L.R.C.P.Lond., D.P.H.Camb., Middlesex Hospital; R. A. D. Daniel, L.R.C.S., L.R.C.P.Ed., L.S.A., St. Mary's Hospital; H. Francis, M.R.C.S.Eng., L.R.C.P.Lond., St. Mary's Hospital; F. J. Worth, M.R.C.S.Eng., L.R.C.P.Lond., St. Mary's Hospital.

#### Examining Board in England by the Royal Colleges of Physicians and Surgeons.

The following gentlemen passed the second examination of the Board in Anatomy and Physiology at a meeting of the examiners on Tuesday, April 12th: G. Phillips, A. Hunnard, J. C. Hilbert, W. Roberts, and R. M. Johnston, students of University College; C. A. Ensor, E. T. Scowby, and B. A. Richmond, of Guy's Hospital; C. R. Watson, F. Morley, R. M. Ellis, and B. B. Tahmisian, of St. George's Hospital; F. S. Tidcombe, of St. George's Hospital and Mr. Cooke's School of Anatomy and Physiology; G. F. S. Genge, and H. Morris, of Westminster Hospital; J. E. G. Calverley, A. W. R. Cochrane, F. M. Burnett, J. F. Bill, and A. H. Morris, of St. Bartholomew's Hospital; W. D. Frazer and R. G. Strange, of St. Thomas's Hospital; A. P. Gibbons, and C. B. Howse, of London Hospital.

Passed in Anatomy only: E. G. Moon, student of St. Mary's Hospital; W. A. Carden, of Guy's Hospital; M. H. C. Palmer, of London Hospital; and A. A. Rogers, of St. Bartholomew's Hospital.

Eight candidates were referred in both subjects, and four in Physiology only.

The following gentlemen passed the second examination of the Board in Anatomy and Physiology at a meeting of the examiners on Wednesday, April 13th: E. Coleman, student of Guy's Hospital; W. Hardcastle and E. H. Fricke, of Charing Cross Hospital; F. J. Coutts, A. H. Gerrard, T. A. Starkey, and J. N. Brown, of University College; N. E. Thomas, of Middlesex Hospital; G. G. Genge and A. C. Swinhoe, of St. Thomas's Hospital; T. B. Bokenham and J. S. Chater of St. Bartholomew's Hospital; H. S. Basden, of London Hospital; and C. Shields, of St. Mary's Hospital.

Passed in Anatomy only: L. H. Y. Stephens and E. Fisk, students of Guy's Hospital; A. C. Bean, of University College; W. F. Adams, W. P. Thomas, and J. J. Spears, of London Hospital; M. A. Cooke, and A. H. Beadles, of St. Bartholomew's Hospital; C. J. Barnes, of King's College and Mr. Cooke's School of Anatomy and Physiology; and J. W. Stokes, of University College.

Passed in Physiology only: W. N. Baron, student of St. Bartholomew's Hospital; and F. A. Phillips, of St. George's Hospital.

Ten candidates were referred in both subjects, two in Anatomy only and ten in Physiology only.

Passed in Anatomy and Physiology on Thursday, April 14th: V. J. Blake, student of University College; D. W. Wiseman, C. W. Young, W. E. Wyborn, and E. C. Montgomery, of Charing Cross Hospital; G. Soilleux, of Melbourne University; J. Currie, G. A. Crace-Calvert, and M. D. Eder, of St. Bartholomew's Hospital; B. J. Mayne, of Middlesex Hospital; E. C. Thurston, of St. Thomas's Hospital; G. U. Smith, H. E. A. Horsford, and P. C. E. Tribe, of King's College; R. L. Norman, of St. George's Hospital; E. H. Read, J. A. Stainsby, and W. Wilson, of London Hospital.

Passed in Anatomy only: J. H. Wigham, student of Yorkshire College, Leeds, and St. Mary's Hospital; M. Wilks, of University College; W. Gilbertson and M. A. Teale, of St. Thomas's Hospital.

Passed in Physiology only: S. A. Francisco, student of King's College; A. R. S. Freeland and G. A. T. Hayden, of London Hospital; W. C. F. Hayward, of Charing Cross Hospital; and A. A. Lewins, of McGill College, Montreal, and Mr. Cook's School of Anatomy and Physiology.

Six candidates were referred in both subjects, two in Anatomy only, and seven in Physiology only.

